# Chronic Kidney Disease and Mortality in Implantable Cardioverter-Defibrillator Recipients

**DOI:** 10.4061/2010/989261

**Published:** 2010-08-03

**Authors:** Aamir Cheema, Tejwant Singh, Manreet Kanwar, Karuna Chilukuri, Viqar Maria, Fareena Saleem, Katrina Johnson, John Frank, Luis Pires, Sohail Hassan

**Affiliations:** Division of Cardiology, Department of Medicine, St. John Hospital & Medical Center, 22101 Moross Road, Cardiac Cath Lab, Detroit, MI- 48236, USA

## Abstract

Incidence of sudden cardiac death (SCD) in end-stage renal disease (ESRD) remains high. Limited data is available about whether implantable cardioverter-defibrillators (ICDs) can prevent arrhythmic death in patients with chronic kidney disease (CKD). The purpose of this retrospective study was to determine the impact of CKD on all-cause and sudden cardiac death in ICD recipients. We evaluated 441 consecutive patients who underwent ICD implantation at our center between 1994 and 2002. We found that mortality rate was higher in patients with eGFR <60 mL/min and those with ESRD on hemodialysis (43%, *n* = 69/162 and 54%, *n* = 12/22, resp.) than in patients with eGFR ≥60 mL/min (23%, *n* = 58/257; *P* < .0005). The SCD rate was also higher in the patients with ESRD (50%) than in CKD patients not on dialysis (10.2%; *P* < .0005). Mortality rate for single-chamber ICDs was 56.8% in comparison with dual-chamber ICDs (38.1%) and for biventricular ICDs (5.0%) (*P* < .0005).

## 1. Introduction

Approximately 4.5% of the adult US population (8 million adults) suffers from CKD [1]. Among the CKD patients, the highest mortality rate is in those who have ESRD on dialysis with an estimate of 224.5 deaths/1000 patient-years [2]. The single largest cause of death amongst ESRD patients on hemodialysis is sudden cardiac death due to arrhythmic mechanism, as 54.6% die of a cardiac arrest and 8.8% die of a cardiac arrhythmia [3]. Furthermore, the rate of cardiac arrest increases progressively in relation to the duration of dialysis therapy [3]. Despite this extremely high arrhythmic mortality rate, currently there is no effective intervention to prevent cardiac arrest in these patients. ICD implantation may decrease risk of sudden cardiac death in this cohort of patients.

Recently, multiple randomized trials have established the benefit of implantable cardioverter-defibrillators (ICDs) in improving survival in high-risk cardiac patients [4]. However, the randomized trials excluded patients with ESRD and included only a minority of patients with mild to moderate CKD [5–8]. Therefore, there is limited data available on the role of ICD in preventing sudden cardiac death in ESRD patients. Also, little is known about the impact of CKD in determining all-cause and sudden cardiac death in the patients who undergo ICD implantation.

The benefit of cardiac resynchronization therapy with ICD as compared to medical therapy alone has been shown in advanced heart failure (HF) patients [9]. However, no data is available on the relative impact of different types of pacing with ICD on long-term survival in the patients with advanced renal failure. The purpose of this study was to analyze the effect of CKD on mortality in ICD recipients.

## 2. Materials and Methods

### 2.1. Patient Population

In this retrospective review, we evaluated consecutive patients who underwent ICD implantation at St. John Hospital from 1994 to 2002. We obtained an approval from the Investigational Board Review committee of St John Hospital and Medical Center for the collection and publication of data. For the purpose of this study, the patients were divided into three groups based on severity of renal dysfunction, derived from KDOQI (Kidney Disease Outcomes Quality Initiative) classification [10]: eGFR ≥ 60 mL/min; eGFR < 60 mL/min; ESRD undergoing hemodialysis.

### 2.2. Study Protocol

The study was approved by the St. John Hospital's Institutional Review Board. The enrolled subjects had ICD implantation based on the following criteria based on different randomized control trials. (1) Nonsustained VT in patients with coronary artery disease (CAD), previous myocardial infarction (MI), left ventricular (LV) dysfunction, and ejection fraction (EF) <35% who had induced sustained monomorphic VT that was nonsuppressible with anti-arrhythmic drug, (Multicenter Automatic Defibrillation Implantation Trial investigation: MADIT) [5]. (2) EF ≤30% in patients with a history of MI (MADIT II) [11]. (3) VF or sustained VT with syncope, or sustained VT with an LVEF <40% and severe symptoms (syncope, near syncope, CHF, angina) suggestive of hemodynamic compromise, (Antiarrhythmics Versus Implantable Defibrillators, AVID criteria) [12]. (4) Syncope of unknown origin in CAD patients, and severe LV dysfunction who had inducible sustained monomorphic VT with hemodynamic compromise at EP study [13]. Estimated measurement of their glomerular filtration rate (eGFR/GFR) was obtained using Cockroft-Gault Formula. Lowest value of serum creatinine was used for calculating the eGFR obtained from the preprocedure blood work closest in time to device implantation. The patients were followed up in the outpatient clinic until September 2004. The mortality information was obtained from a variety of sources including the records from the hospital, physician office, nursing home, and the social security death index. The information was extracted using a structured form. Mode of death was classified as cardiac-sudden (arrhythmic), cardiac nonsudden, noncardiac, and unknown. Sudden cardiac death (SCD) was defined as a sudden unexpected pulseless condition of likely cardiac etiology. If unwitnessed, SCDs were those in which patients were found dead within 24 hours of having last been seen alive and in normal state of health [14]. Each patient death was reviewed by a cardiologist and a nephrologist. Patients were carefully evaluated for any history of withdrawal from dialysis prior to their death and none of the 12 patients who had as SCD were removed from dialysis nor missed a dialysis session prior to their cardiac arrest.

### 2.3. Statistical Analysis

Continuous variables were expressed as mean ± standard error (SE) and compared using unpaired *t*-test or ANOVA, as appropriate. Categorical variables were expressed as frequency (%) and compared by Chi-Square analysis. Kaplan-Meier analysis was conducted to evaluate survival differences between groups. Multivariate logistic regression was performed to determine predictors of mortality. A *P* value of <.05 was considered statistically significant. All statistical analyses were performed using SPSS statistical software (SPSS 12.0, Chicago, IL).

## 3. Results

The clinical characteristics of the 441 patients (340 men (77%); mean age 66.8 ± 0.6 (±SE) years), based on the patient's level of kidney dysfunction, are shown in Table 1. Not unexpectedly, the age of the three groups differed (ANOVA, *P* < .0005). Patients with GFR < 60 mL/min were older than each of the other groups (Scheffe post hoc analysis, *P* < .0035), and the proportion of Caucasians was higher (Chi Square, *P* = .041). Diabetes was most prevalent in patients on hemodialysis (*P* = .028). Serum magnesium and potassium were highest in patients undergoing dialysis (ANOVA, *P* < .0005). A higher proportion of patients on hemodialysis were receiving amiodarone (40.9%; Chi Square, *P* = .012), possibly because many of these patients were noted to have atrial fibrillation (*P* = .049). Patients on hemodialysis were receiving ACE-Is/ARBs at the lowest rate (59.1%; *P* = .025).

### 3.1. Long-Term Survival Analysis

**Figure 1:**
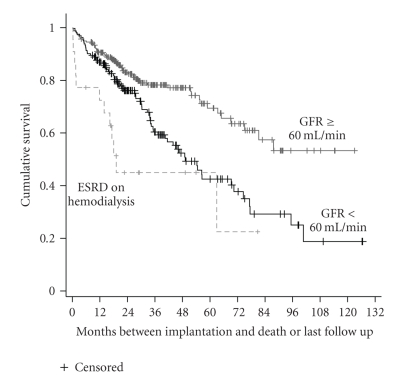
Kaplan-Meier (K-M) survival analysis based on stages of kidney dysfunction. The Log Rank statistic for the K-M Survival analysis was 25.71 (*P* < .00005). For patients with GFR < 60 mL/min (*n* = 162), mean survival time (±SE) was 60.3 ± 4.7 months (CI 51.0, 69.5) and median survival time was 49.2 ± 5.8 months (CI 37.7, 60.7). For patients with GFR ≥ 60 mL/min (*n* = 257), mean survival was 85.9 ± 4.3 months (CI 77.4, 94.3); since survival rate did not drop below 50% during the observation period, no median could be calculated. For ESRD patients on hemodialysis (*n* = 22), mean survival was 37.8 ± 7.7 months (CI 22.8, 52.8) and median survival was 19.1 ± 1.8 (CI 15.6, 22.7).

The overall survival was 85.9 ± 4.3, 60.3 ± 4.7, and 37.8 ± 7.7 months for those with GFR ≥ 60 mL/min, those with GFR of <60 mL/min, and ESRD patients on hemodialysis, respectively (Log rank statistic 25.71; *P* < .00005). Shown in Figure 1 are the Kaplan-Meier survival curves for these three groups. The death rate was highest in the patients on hemodialysis (54.5%), followed by patients with GFR < 60 (42.6%), and lowest in those with GFR ≥ 60 (22.6%; Chi Square, *P* < .0005). Median survival time was 49.2 months for patients with GFR ≥ 60 mL/min and 19.1 months for patients on hemodialysis.

**Figure 2:**
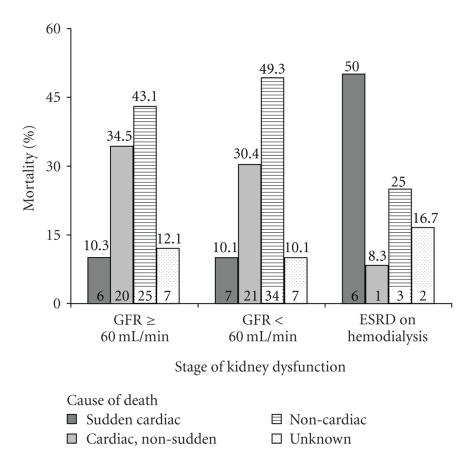
Cause-specific mortality according to varying levels of renal dysfunction. For the 3 categories of renal dysfunction, cause-specific mortality is depicted. As expected, sudden cardiac death was the major cause of death in ESRD patients on dialysis (50.0% versus 10.1% (GFR < 60 mL/min) versus 10.3% (GFR ≥ 60 mL/min), Chi Square *P* = .010). Number at the top of each bar is the mortality rate; number within the bar is the *n* per group. The Unknown category was reserved for those patients whose cause of death could not be determined. Thus, adding the numbers within the bars for three groups will not equal the *n* given in Table 1.

Figure 2 shows the incidence of different modes of death in the three groups. The rate of sudden cardiac death was significantly higher for patients with ESRD on hemodialysis (50.0%) than for those with GFR < 60 and GFR ≥ 60 mL/min (10.1% versus 10.3%, resp.; *P* = .001).

### 3.2. Univariate Analysis

The univariate predictors of mortality were age at ICD implantation, GFR, serum creatinine and magnesium, Caucasian race, taking beta blockers, taking ACE-Is/ARBs, reason ICD was implanted, and type of ICD (Table 2).

### 3.3. Multivariate Analysis

The results of multivariate logistic regression analysis, given in the right-hand columns of Table 2, revealed that age, decreased eGFR, diabetes mellitus, the absence of beta blockers, absence of ACE inhibitors/ARBs, and the type of ICD were independent predictors of all-cause mortality. As compared to the biventricular ICD, single-chamber and dual chamber ICDs were independently associated with decreased survival. The highest odds ratio was generated for the single chamber ICD (10.499; 95% CI 3.238–34.045). The data was adjusted for gender, ischemic heart disease, ejection fraction, primary or secondary prevention, amiodarone, race, HTN, atrial fibrillation, magnesium, potassium, creatinine, defibrillation threshold 20 joules and above, QRS over 150 mSec, and type of revascularization.

## 4. Discussion

The main finding of the study is that chronic kidney disease is independently linked to increased all-cause mortality in ICD recipients. Second, the incidence of SCD in this cohort of patients is significantly high in those with ESRD on hemodialysis as compared to those with less advanced CKD. Third, the clinical factors which independently predict increased mortality in the patients who undergo ICD implantation include increased age, decreased estimated GFR, absence of beta blockers, diabetes mellitus, absence of ACE/ARBs, and type of ICD. The absence of beta-blockers and ACE inhibitors identifies patients with persistent hypotension, which highlights severe end-stage cardiomyopathy. These observations extend the previous data that has shown that these variables are associated with mortality in HF patients [15]. The survival benefit of ICD type is unlikely to be due to chance, since the proportion of each type implanted in the 3 kidney function categories did not differ (*P* = .112). 

This study confirms previous findings that the commonest mode of death in ESRD patients is SCD [3]. Bleyer et al. reported that the incidence of sudden cardiac death increases by 50% on Monday for patients dialyzing on Monday, Wednesday, and Friday [16]. Despite this extremely high incidence of arrhythmic deaths in this population, there are limited effective preventive strategies available. Recently, data from multiple, large-scale, randomized-controlled trials have shown that ICDs are effective in reducing mortality in high-risk cardiac patients [5–8]. However, all the major trials excluded patients with advanced CKD or ESRD [5–8]. At this point, majority of cardiomyopathy patients with advanced CKD or ESRD are undergoing ICD implantation without any substantial evidence of survival benefit. Since CKD is a very strong and independent predictor of mortality, the risk-benefit ratio of an invasive and expensive therapy such as ICD needs to be specifically studied in CKD patients, before extrapolating the findings of the ICD trials to this population. 

 Herzog et al. used the Medicare database to retrospectively study over 6000 ESRD patients surviving cardiac arrest. They reported a 42% reduction of death risk in the patients who received ICDs (as compared to those who did not) over a five-year period [17]. This data supports the use of ICDs in the secondary prevention of sudden cardiac death in ESRD patients. However, the majority (85%) of the ESRD patients hospitalized after the cardiac arrest die in one year [10]. The current study showed a higher mortality rate in patients who had ICD implantation for secondary prevention as compared to those with primary prevention ICD. These observational findings support the hypothesis that implanting an ICD for primary prevention in a cardiomyopathy patient, already on the continuum of CKD, may be more effective before they have had a cardiac arrhythmia or arrest (Table 2). 

Based on the findings of our study, further prospective studies will define the role of the ICD implantation and whether a certain type of ICD will benefit renal failure patients more in prevention of sudden cardiac death. The results are consistent with the recent report of Wase et al. who studied 95 patients who underwent ICD implantation and found higher mortality in the patients with an eGFR < 60 cc/min as compared to those with an eGFR ≥ 60 cc/min [18]. 

The exact causes of this alarmingly high total and arrhythmic mortality in CKD patients are multifactorial. First, CKD alone is an independent risk factor for coronary artery disease (CAD) [19]. Second, the traditional cardiovascular risk factors, that is, hypertension (which may be accompanied by left ventricular hypertrophy), smoking, diabetes, metabolic syndrome, dyslipidemia, and older age, are highly prevalent in CKD patients [20, 21]. Third, there are possible additional risk factors that are relatively unique to patients with moderate to severe CKD. These include decreased excretion of uremic toxins, anemia, increased calcium intake, abnormalities in bone mineral metabolism, hyperhomocysteinemia, and/or an “increased inflammatory-poor nutrition” state [22]. And finally, CKD patients have a higher propensity towards increased sympathetic activity and arrhythmias [23]. Higher defibrillation thresholds (DFTs), in CKD patients receiving ICD, have been reported as compared to those without CKD [18]. Among the patients with higher DFTs who undergo ICD implantation, the majority die of arrhythmias [24]. This observation might explain the high incidence of arrhythmic deaths in ESRD patients who undergo ICD implantation, despite the presence of ICDs. 

This survival data after adjusting for potential confounders in multivariate analysis suggests that compared to single chamber ICDs, dual chamber and biventricular ICDs (patients receiving cardiac resynchronization therapy) are independently associated with improved survival (Table 2). The benefits of dual chamber pacing (i.e., pacing both the right atrium and/or right ventricle) compared to single chamber (i.e., right ventricle) could be explained by the following potential benefits associated with dual chamber pacing: (1) presence of chronic atrial fibrillation possibly affecting survival in recipients of single chamber ICDs, (2) the detrimental effect of chronic right ventricle (RV) pacing (which is more likely to be avoided or minimized with AAI pacing mode in a dual-chamber ICD as compared to a single-chamber ICD), triggering mechanical dyssynchrony between the left ventricle and right ventricles [25], and (3) in some studies, atrial pacing has reduced the frequency of atrial fibrillation [26]. Biventricular ICD was identified as a stronger predictor of improved survival, as compared to dual-chamber ICD. It is likely that this difference is due to the detrimental effect of RV pacing, which may cause dys-synchrony between the ventricles [25]. RV pacing causes the RV to contract before the left ventricle (LV), simulating the effects of left bundle branch block (LBBB). Pacing-induced loss of ventricular synchrony worsens heart failure (HF) [27]. Correction of ventricular dyssynchrony in patients with HF via biventricular pacing (or cardiac resynchronization therapy) improves patient survival and other clinical outcomes [28].

The study had the following limitations. First, part of the study data was collected retrospectively. Second, although every effort was made to collect the lowest serum creatinine level available in the absence of acute renal failure, creatinine may not have been at baseline in some patients. And third, intracardiac electrogram data was not available to confirm the mode of death. However, a clinically validated definition of sudden cardiac death was used [14].

## 5. Conclusions

In patients undergoing ICD implantation, the stage of kidney dysfunction is a strong predictor of mortality. The ESRD patients on hemodialysis had a higher incidence of sudden cardiac death (50%) than CKD patients not on dialysis (10.2%). Other predictors of mortality include increased age, ICD type, diabetes mellitus, absence of beta blocker, and absence of ACE inhibitors/ARBs. ICD insertion for primary prevention was more effective in averting fatal events than insertion for secondary prevention in this nonrandomized study. This study underscores the need for conducting large-scale, randomized, controlled trials, to prospectively evaluate the benefits and risks of ICD therapy in patients with cardiomyopathy and kidney dysfunction.

## Figures and Tables

**Table 1:** Characteristics of the study population. Groups based on eGFR or being on hemodialysis.

	GFR		ESRD on	
	≥60 mL/min	<60 mL/min	hemodialysis	
	(*n* = 257)	(*n* = 162)	(*n* = 22)	*t*-test or *χ* ^2^
Characteristics	Mean ± SE or % (*n*)			*P*
Age (yr)	62.6 ± 0.8	73.7 ± 0.7	65.0 ± 2.9	<.0005
Male Gender	78.6 (202)	75.9 (123)	68.2 (15)	NS
Caucasian Race	72.0 (185)	82.7 (134)	72.7 (16)	.041
Hypertension	70.8 (182)	70.8 (114)	90.9 (20)	NS
Diabetes Mellitus	28.8 (74)	27.2 (44)	54.5 (12)	.028
Ischemic Heart Disease	77.0 (151)	83.2 (109)	88.9 (16)	NS
Ejection Fraction (%)	26.6 ± 0.8	25.2 ± 0.8	25.0 ± 2.6	NS
eGFR (mL/min)	93.6 ± 2.0	44.7 ± 0.8	10.3 ± 0.4	<.0005
Creatinine (mg/dl)	1.02 ± 0.02	1.52 ± 0.04	7.14 ± 0.30	<.0005
Magnesium (mg/dl)	1.75 ± 0.02	1.77 ± 0.02	2.01 ± 0.5	<.0005
Potasium (mEq/L)	4.16 ± 0.03	4.28 ± 0.43	4.68 ± 0.14	<.0005
Amiodarone	15.6 (40)	18.5 (30)	40.9 (9)	.012
Atrial fibrillation	19.8 (51)	28.4 (46)	36.4 (8)	.049
DFT > 20 Joules	35.3 (88)	27.7 (43)	66.7 (2)	.129
Beta-blockers	46.7 (120)	40.7 (66)	63.6 (14)	.103
ACEIs/ARBs	78.6 (202)	69.1 (112)	59.1 (13)	.025
Reason for ICD				NS
Primary prevention	51.3 (132)	56.7 (92)	54.5 (12)	
Secondary prevention	48.8 (125)	43.5 (70)	45.4 (10)	
Type of ICD				.112
Single Chamber	30.0 (77)	39.5 (64)	36.4 (8)	
Dual Chamber	59.1 (152)	45.7 (74)	54.5 (12)	
Biventricular	10.9 (28)	14.8 (24)	9.1 (2)	

NS: *P* > .05.

**Table 2:** Mortality Predictors in Univariate and Multivariate Analyses.

	Alive	Dead	*t*-test or		
	(*n* = 302)	(*n* = 139)	*χ* ^2^	Logistic Regression	
Predictors	Mean ± SE or % (*n*)		*P*	OR	95% CI
Age (yr)	65.2 ± 0.7	70.3 ± 0.9	<.0005	1.034	1.005–1.064
eGFR (mL/min)	78.1 ± 2.2	57.0 ± 2.6	<.0005	.986	.975–.997
Creatinine (mg/dl)	1.38 ± 0.07	1.80 ± 0.14	.008		
Magnesium (mg/dl)	1.75 ± 0.01	1.81 ± 0.03	.019		
Caucasian Race	73.2 (221)	82.0 (114)	.044		
Diabetes Mellitus	27.2 (82)	34.5 (48)	.114	2.094	1.129–3.883
Beta-blockers	53.3 (161)	28.1 (39)	<.0005	.342	.190–.614
ACEIs/ARBs	78.5 (237)	64.7 (90)	.002	.515	.269–.985
Reason for ICD			.005		
Primary prevention	57.9 (175)	43.8 (61)			
Secondary prevention	42.1 (127)	56.2 (78)			
Type of ICD			<.0005		
Single Chamber	23.2 (70)	56.8 (79)		10.499	3.238–34.045
Dual Chamber	61.3 (185)	38.1 (53)		4.232	1.326–13.505
Biventricular	15.6 (47)	5.0 (7)		1.0	
